# Nonlytic Egress and Transmission in the Virus World

**DOI:** 10.1146/annurev-biochem-052521-120140

**Published:** 2025-06

**Authors:** Nihal Altan-Bonnet, Mamata Panigrahi

**Affiliations:** Laboratory of Host-Pathogen Dynamics, National Heart, Lung, and Blood Institute, National Institutes of Health, Bethesda, Maryland, USA

**Keywords:** virus, egress, transmission, nonlytic, en bloc, extracellular vesicle

## Abstract

Viruses must egress from the cells in which they have replicated to spread and propagate. Historically, viruses have been classified into enveloped and nonenveloped forms: Enveloped viruses exploit cellular membrane-trafficking pathways to egress while maintaining cell integrity, and nonenveloped viruses, i.e., those lacking a membrane around their capsids, lytically egress. Here, we make the compelling case that all animal and plant and many archaeal and bacterial viruses egress through nonlytic pathways. Most of these nonlytic pathways can be separated into those that enable viruses to spread without leaving the confines of cell bodies and those that traffic them to the extracellular space in enveloped membrane-bound forms. Nonlytic egress pathways bestow viruses with distinct transmission advantages including high multiplicity of infection, quality control over transmitting infectious units, and evasion of innate and adaptive antiviral immune defense mechanisms.

## 1. INTRODUCTION

Living cells provide the building blocks for viral replication and propagation including the translation machinery, lipids, nucleotides, amino acids, and energy. A critical event in the viral life cycle is the egress of newly replicated and assembled viral particles, allowing their spread to other host cells. The cellular pathways exploited or altered during egress, whether the cell is left intact or lysed, the quantity and form in which the virus egresses, and whether the immune system is alerted are all major factors determining the success of viral transmission.

The field of virus classification evolved hand in hand with the development of the electron microscope (1). The first electron micrographs were taken of a plant virus, the tobacco mosaic virus (2). This new technology, which enabled visualization at the nanometer scale and the development of various heavy metal specimen-staining methods, soon led to the systematic visualization of viruses from samples obtained from cell culture and from the feces, sputum, and other secretions of infected individuals. From these images emerged one of the major ways by which viruses have since been classified: as enveloped, i.e., with a membrane surrounding their nucleocapsid, or nonenveloped, i.e., with just the nucleocapsid (3). Viral families classified as enveloped include *Flaviviridae* [e.g., Dengue virus (DENV), West Nile virus, Langat virus, hepatitis C virus (HCV)], *Retroviridae* [e.g., human immunodeficiency virus (HIV)], *Coronaviridae* [e.g., severe acute respiratory syndrome (SARS) viruses, Middle East respiratory syndrome virus (MERS)], *Filoviridae* (e.g., Ebola and Marburg viruses), *Herpesviridae* (e.g., herpes simplex virus, Epstein–Barr virus), and *Poxviridae* (e.g., smallpox and vaccinia viruses), and nonenveloped viral families include *Picornaviridae* [e.g., poliovirus, coxsackievirus, rhinovirus, enterovirus 71 (EV-71), hepatitis A and E viruses (HAV and HEV)], *Caliciviridae* (e.g., norovirus), *Polyomaviridae* (e.g., SV40), *Adenoviridae*, *Parvoviridae*, *Birnaviridae*, and *Sedoreoviridae* [e.g., rotavirus, reovirus, bluetongue virus (BTV)], as well as the vast majority of plant and bacterial viruses and some archaeal viruses.

This simple morphological classification led to one of the major dogmas persisting in virology, that nonenveloped viruses must lyse their host cells to egress and transmit, while enveloped viruses are nonlytically secreted by budding into host membrane-trafficking pathways. While cell death and lysis can still occur with the latter [and do with many enveloped viruses, including Ebola, DENV, SARS coronavirus 2 (SARS-CoV-2), and influenza], they are not considered prerequisites for egress as they are for nonenveloped viruses. Yet, lytic egress for any virus is not an optimal strategy for propagation and transmission. Firstly, lysis results in the release of inflammatory cytoplasmic molecules with damage-associated molecular patterns, complement proteins, cytokines, and chemokines, all of which activate immune cells, trigger inflammatory responses by binding to pattern-recognition receptors, and further amplify the inflammatory response by forming pores in nearby cells (4–12). Secondly, cell death, with its disintegration of cellular organelles, loss of membrane potential, and disruption of cellular metabolism, limits the amount of viral progeny produced (13). Thirdly, lysis leads to indiscriminate egress, including that of nonreplicative empty viral capsids that can exacerbate the immune response by stimulating antibody production (14–16). Lastly, keeping cells alive during infection can be a means for some viruses to set up persistent or latent infections (17–19). Therefore, it is not surprising that viruses have developed numerous mechanisms to delay or even entirely avert cell death and lysis, including preventing the initiation of death pathways and inhibiting death and lysis effectors such as Bax, Bcl-2, caspases, and membrane pore-forming molecules like gasdermin D (20, 21).

**Figure 1:**
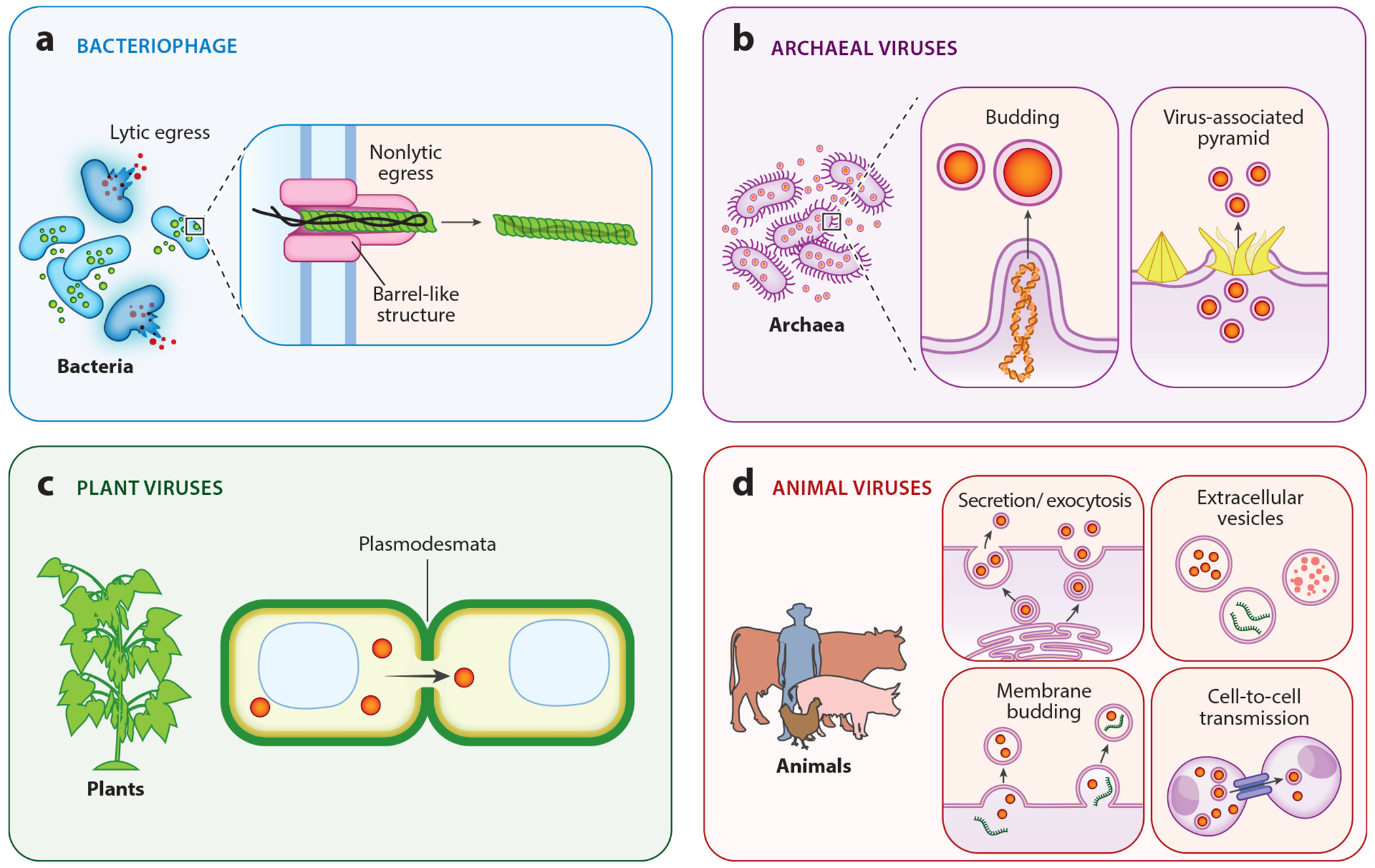
Nonlytic egress pathways adopted by viruses have evolved alongside their hosts, resulting in diverse and multifaceted mechanisms. (*a*) Generally, bacteriophage egress is lytic. However, certain bacteriophages induce the formation of barrel-like structures to exit the host cell without causing lysis. (*b*) Similarly, archaeal viruses employ nonlytic egress mechanisms, such as membrane budding or the induction of specialized structures known as virus-associated pyramids (VAPs). The opening of these VAPs facilitates viral exit from the host cells. (*c*) Plasmodesmata in plants allow viruses to move and spread among cells. (*d*) In animal cells, nonlytic viral egress can take place through the biosynthetic secretory pathway, the endo/lyso/autophagosomal exocytic pathways (which can result in virus transport in extracellular vesicles), and membrane budding, as well as cell-to-cell transmission through channels, nanotubes, and syncytia.

Here, we propose that nonlytic egress is the dominant and most functionally relevant form of egress in the virus world, with the majority of animal and plant viruses exploiting essentially two major nonlytic egress pathways for transmission: one by which viruses traffic out of cells to the extracellular space in enveloped forms, either as membrane-cloaked individual viruses or membrane-cloaked clusters of viral particles, and one in which they remain intracellular while spreading from cell to cell (Figure 1). We argue that nonlytic egress has distinct advantages including increasing the chances of transmitting infectious genomes, increasing the multiplicities of infection, and helping evade innate and adaptive antiviral immune responses.

## 2. NONLYTIC EGRESS IS WIDESPREAD IN THE NONENVELOPED VIRUS WORLD

The field of modern virology got its start with the pioneering studies of the bacteriophage lifecycle by Félix d’Herelle (22) and Max Delbrück (23), which impressed very early upon this burgeoning field the cytopathic nature of viral infections and the need for cells to lyse for newly propagated viruses to egress. One of the tools developed to quantify bacteriophages, the plaque assay, is indeed a direct extension of the largely cytopathic nature of bacteriophage propagation (though there are nonlytic bacteriophages, such as the *Inoviridae*, as discussed in Section 6). For this reason, the early electron micrographs of animal and plant viruses by Ruska (24), Brenner & Horne (25), and others (26–30) were also carried out with biological samples collected at late stages of the viral infection, when cytopathic effects dominate. This raises the question of whether those micrographs of extracellular viruses were truly egressed viruses or viral forms that were indiscriminately ejected from lytic cells because of viral stress and/or killing by the immune system. Indeed, reviewing the literature of the past 70 years reveals many overlooked reports documenting nonlytic egress of so-called nonenveloped viruses. Ackermann & Kurtz (31) observed that poliovirus could persist in HeLa cell cultures while the cells underwent mitosis. Takemoto & Habel (32) reported persistent infection by coxsackievirus A9 of HeLa cells. Carson and colleagues (33) observed multiple echovirus strains egressing from monkey cell lines with either a complete lack or a delayed appearance of plaques (5–10 days postinfection). Compans and colleagues (34) found poliovirus to be released in a polarized manner from enterocytes.

Molecular tools to monitor cell lysis and viral egress have also challenged the prerequisite nature of cell lysis during viral egress for the purported nonenveloped virus. Membrane-impermeable small molecules like Trypan blue and lucifer yellow are useful for gauging the intactness of the plasma membrane and cell viability during viral egress (see Supplemental Material). One of the earliest direct measurements of the permeability of the plasma membrane relative to egress of a nonenveloped virus was carried out using Trypan blue on SV40 polyomavirus–infected cells (35). Compans and colleagues (35) found that the plasma membrane remained resistant to Trypan blue uptake as late as 24 h after the completion of SV40 egress. Similar assays were carried out on other polyomaviruses as well as the *Picornaviridae* poliovirus, coxsackievirus, and rhinovirus, and in all cases, viral egress was reported to take place many hours prior to disruption of the plasma membrane and cell death (36–38).

Trypan blue exclusion assays also revealed that the *Sedoreoviridae* family of viruses, which includes rotavirus, reovirus, and BTV, all begin egress prior to cell lysis. Indeed, some rotavirus strains, when inoculated into human cholangiocytes, completely averted cell killing and lysis for days after the start of egress (39). Similar assays carried out on reoviruses and BTV have also reported nonlytic egress, with >90% of BTV egress occurring in the absence of any cell lysis (40, 41). Likewise with murine and human noroviruses in the *Caliciviridae* family; rhesus enteric calicivirus; the *Astroviridae* family members VA-1, MLB, and HuAstV-8; and the *Birnaviridae* family member infectious bursal disease virus, direct measurements of plasma membrane intactness revealed egress to take place hours before any changes in cell plasma membrane permeability could be detected (39, 42–47). Furthermore, a recent report showed that depleting the host plasma membrane pore-forming molecule Ninjurin-1, which had been previously thought to aid murine norovirus (MNV) propagation by facilitating lysis of the plasma membrane, reduced MNV egress only by <1 log_10_ decrease (48). Investigations into the egress of a *Parvoviridae* family member, minute virus of mice, also revealed that newly assembled progeny virions left the infected rat cells nonlytically, and this enabled the cells to keep proliferating, leading to more viral production (49). Some strains of the *Adenoviridae* family exhibited only nonlytic egress in vivo and were resistant to the FDA-approved drug Nelfinavir, which appeared to be able to inhibit only lytic transmission (50). Lastly, the majority of plant viruses, as well as many archaeal and some bacterial viruses, have been shown to transmit nonlytically (Figure 1) (51–57).

Given that so many nonenveloped viruses egress nonlytically, why is cell lysis so frequently associated with these viruses? One possibility is that these viruses, even though they themselves do not use lysis as an egress mechanism, nevertheless trigger the pathways leading to cell death and lysis earlier than enveloped viruses do. Challenging this, however, are findings with rotavirus, poliovirus, coxsackievirus, HAV, echovirus, polyomaviruses, caliciviruses, adenoviruses, and many others, demonstrating that the same strain of virus can lead to lysis in one cell type and not another (34, 37, 39, 47, 58). MA104 epithelial cells infected with rotavirus strain SA-11 inevitably die by lysis even though SA-11 egress is nonlytic and begins hours prior. In contrast, human cholangiocytes infected with SA-11 remain viable while virus replicates and egresses (39). Although type-1 Mahoney poliovirus egresses nonlytically from Caco-2, Vero, and different HeLa-derived cell lines, the timing of cell death and lysis differs vastly among these cell types (31, 34, 37). Thus, the natural tendency and timing of a cell losing its viability likely has less to do with the infecting virus strain than with the inherent sensitivity of its death and lysis signaling pathways to viral infection. All in all, experiments that carefully monitor viral egress in conjunction with cell lysis point to significant time delays between egress and cell lysis. Hence, the textbook observations of viruses in nonenveloped states might be experimental artifacts of the timing of the collected samples (biased by the need to obtain isolated viruses for electron microscopy). In turn, this challenges our understanding of the functional relevance of host-cell lysis for the life cycle of the viruses.

## 3. NONLYTIC TRAFFICKING OF VIRUSES AND INFECTIOUS VIRAL COMPONENTS TO THE EXTRACELLULAR SPACE IN MEMBRANE CLOAKS

**Figure 2:**
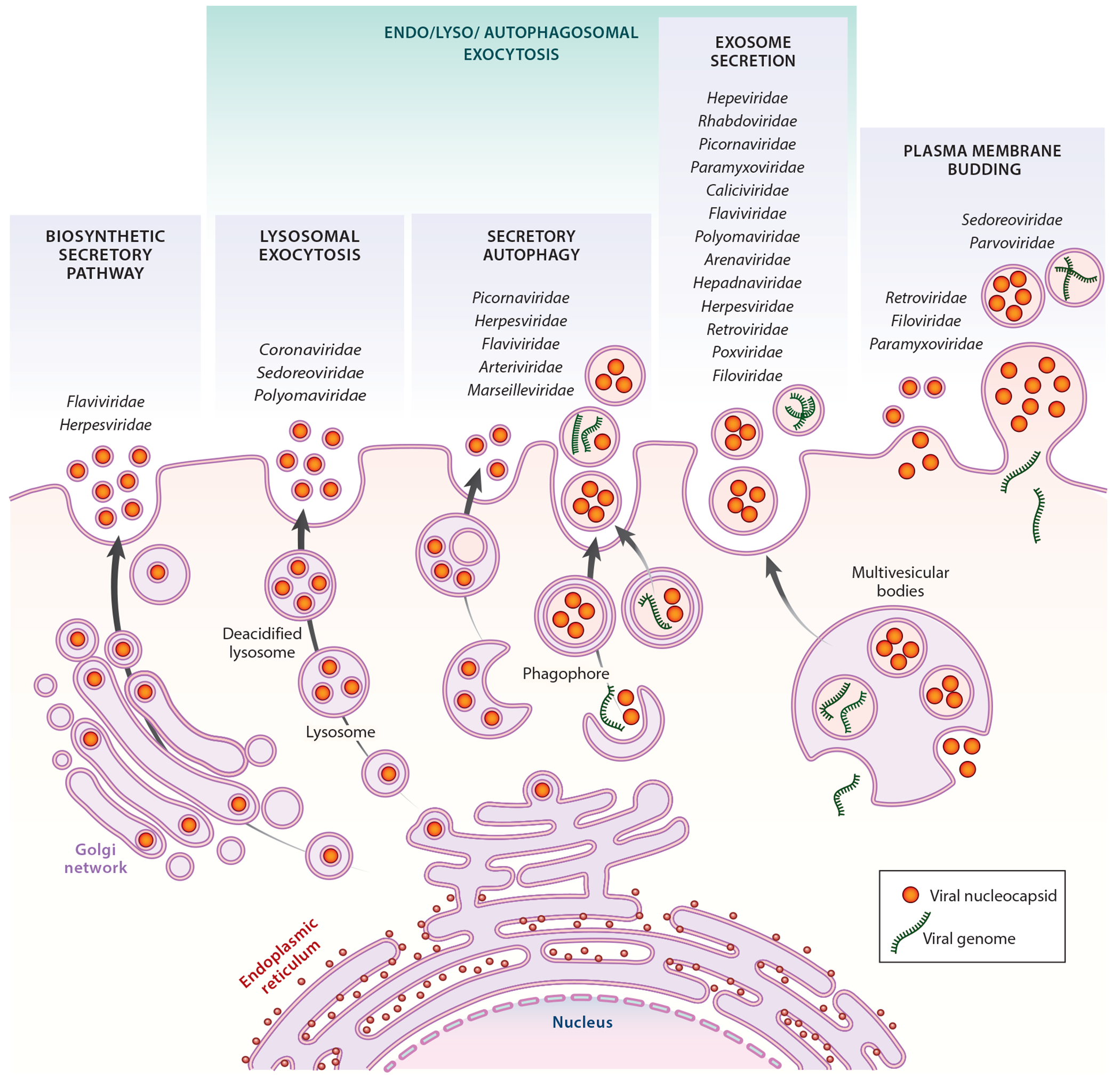
Cellular exocytic membrane-trafficking pathways enable viruses to egress nonlytically and enveloped by a membrane. The biosynthetic secretory pathway and the endo/lyso/autophagosomal exocytic pathways, which include secretory autophagy and lysosomal and multivesicular body (MVB) exocytosis, and plasma membrane budding, including the formation of microvesicles, all provide nonlytic paths of egress for both conventional enveloped and historically so-called nonenveloped virus families. Viral nucleocapsids either individually bud or become cloaked en bloc through these membrane-trafficking pathways. These pathways also facilitate the egress and transmission of cytoplasmic naked viral genomes and replication complexes.

Given the experimental observations reviewed above, how can nonenveloped viruses egress to the extracellular space without compromising the integrity of the host cell? Recent studies using a combination of better sample preparation, high resolution light-based imaging, and electron microscopy have revealed that they do so by entering the same membrane-trafficking pathways used by enveloped viruses and emerging similarly in an enveloped state, cloaked by a membrane (Figure 2).

### 3.1. Biosynthetic Pathway

The biosynthetic pathway is a major trafficking pathway that transports newly synthesized proteins and lipids via vesicle carriers from the endoplasmic reticulum (ER) to the extracellular space, passing through the Golgi and *trans*-Golgi network (TGN) in between. It is also a major nonlytic egress pathway for *Flaviviridae* (DENV, Langat, West Nile, HCV) (59–61), and *Herpesviridae* (62) family members. Typically the encapsidated viral genomes enter the pathway by budding into the ER (e.g., *Flaviviridae*) (63, 64) or the Golgi and TGN (e.g., *Herpesviridae*) (65, 66), acquiring a membrane cloak studded with viral proteins that help the viruses engage with their cognate receptors on the next host cell, and begin a new round of infection. Viruses egress via this pathway as enveloped individual particles. These particles may remain thus or aggregate, which can enhance their multiplicity of infection (discussed in Section 8) (67).

### 3.2. Multivesicular Body Exocytosis

Many viruses harness multivesicular bodies (MVBs) to traverse from the cytoplasm to the extracellular space and egress nonlytically (39, 68–76). MVBs originate from endosomal organelles, which undergo invagination to give rise to MVBs whose lumen consists of vesicles carrying cytosolic cargo (77–79). Cargo sorting into the intraluminal vesicles generally involves the selective incorporation of specific membrane proteins, lipids, and soluble cytosolic cargo through the sequential actions of the endosomal sorting complexes required for transport (ESCRT) protein machinery. The sorted cargo can be degraded, if the MVBs fuse with lysosomes, or released into the extracellular environment, if the MVBs fuse with the plasma membrane. These released MVB intraluminal vesicles, termed exosomes and typically 50–200 nm in size, enable both short- and long-distance en bloc delivery of cytosolic cargo between cells (77–79). While the precise mechanisms targeting exosomes to cells and tissues are unclear, the exosomal membranes contain fusogenic proteins, which are often of retroviral origin, like syncytin, and facilitate fusion and delivery of vesicle contents to target cells (80–82).

As MVBs are late endosomal compartments that originate in part from the plasma membrane, it is not surprising that many viral families, including *Hepadnaviridae* (e.g., hepatitis B virus), *Retroviridae, Filoviridae, Paramyxoviridae, Arenaviridae*, and *Rhabdoviridae*, that bud from the plasma membrane also bud into MVBs to egress nonlytically, the topology of egress being equivalent (83–88). Many of these family members do so by having envelope proteins with late (L)-domain sequences that are distributed throughout both the plasma and MVB membranes, which enable interactions with host ESCRT machinery (86, 89–92). This leads to the sorting and envelopment of the viral nucleocapsids into the lumen of the MVB, with the intraluminal vesicles now becoming individual enveloped viral particles. Once the MVB fuses with the plasma membrane, these particles egress to the extracellular environment.

So-called nonenveloped viruses also exploit MVBs to egress nonlytically in membrane-cloaked enveloped forms. Lemon and colleagues (69, 93) were first to report this, showing HAV (*Picornaviridae*) has two infectious forms: naked nucleocapsids, which are primarily found in feces, and nucleocapsids inside exosomes, which are found in the blood. HAV nucleocapsids egress from liver cells nonlytically, in exosomes, as they are sorted into MVBs due to their capsid VP2 proteins that contain multiple L-domain sequences (93). While the exosome membrane remains intact in the blood, it is stripped by bile acids as the HAV passes from the liver to the intestinal system. HEV (*Hepeviridae*) is also released nonlytically and in a polarized manner in exosomes originating from MVBs. This is dependent on viral ORF3 (94) and mediated by its L-domain sequences interacting with the ESCRT-I component TSG101 (70). Noroviruses (*Caliciviridae*) and polyomaviruses (*Polyomaviridae*) have also been shown to release nonlytically from cells inside MVB-derived exosomes, and the polyomavirus JCPyV major capsid protein VP1 contains the L-domain YPX_3_L motifs (95). Their egress can be blocked with inhibitors of the MVB–plasma membrane fusion pathway such as GW486 or Rab27 depletion (39, 96). These virus-containing exosomes contain typical MVB proteins and lipid markers such as tetraspanins (CD63, CD81, and CD9), flotillin-1, Annexin V, and TSG101 and are enriched in phosphatidylserine (PS), sphingomyelin, and lysobisphosphatidic acid lipids, which enhance exosome stability in the body, e.g., by fending off environmental assaults (39, 97–102).

Given that MVBs provide a nonlytic route for cytosolic material to cross the plasma membrane, it is not surprising that they can also shuttle naked viral genomes out of the cell. Clusters of HCV, Langat virus, DENV, Zika virus, West Nile virus, and HIV genomes have all been reported to be secreted from cells inside exosomes, both in vitro and in vivo, and the secreted exosomes are infectious (103–108). These exosomes can effectively deliver the viral genetic material to new host cells, allowing the virus to initiate replication de novo and spread infection, essentially acting as a Trojan horse by utilizing the natural cellular mechanisms of exosome uptake and fusion with target cells, as well as evading immune detection. HCV genome–containing exosomes can be isolated from the sera of chronically HCV-infected patients as well as from the culture media of infected cultures of liver cells (103, 109, 110). DENV-infected mosquito cells can secrete AalCD9^+^ and AalCD81^+^ (arthropod orthologs of human CD9 and CD81) exosomes containing full-length viral genomic RNA and viral proteins, and these exosomes are able to infect naive mosquito and mammalian cells and enhance DENV transmission (107, 111). Langat and West Nile viruses have also both been shown to egress as clusters of full-length naked viral genomes (a mix of both positive and negative RNA strands) via exosomes from both insect and human cells. These exosomes are infectious and can efficiently transmit the viral genomes to human keratinocytes and endothelial cells (105). Although the exact mechanisms by which nucleic acids are recognized by the ESCRT machinery remain to be elucidated, exosomes may expand tissue tropism, bypassing virus particle–binding receptors and instead letting naked viral genomes enter the cytoplasm through fusion of the exosome with target cell membranes.

### 3.3. Secretory Autophagy

Autophagy is generally considered a degradative pathway by which proteins, lipids, and even whole organelles can be recycled to benefit the cell in times of starvation and other stresses. The double-membraned autophagosome structure, or phagophore, typically originates from ER-derived membranes and sequesters cellular materials through the Atg8/LC3 family of cargo receptors (e.g., LC3, GABARAP, GABARAPL1) before fusing with lysosomes (112). *Picornaviridae* family members, including poliovirus, coxsackieviruses, rhinoviruses, EV-71 and EV-D68, all exploit a version of the autophagy pathway, termed secretory autophagy, for nonlytic egress (36, 37, 113–120). Here autophagosomes capture the cytosolic newly assembled nucleocapsids and then fuse with the plasma membrane, releasing these virions to the exterior milieu inside an extracellular vesicle, i.e., the inner membrane of the autophagosome (37, 119). Both secretory and canonical degradative autophagy rely on common machinery including mTOR, ULK, Beclin-1, VPS34, and LC3, and depleting any of these components inhibits both pathways (121–128). In degradative autophagy, autophagosomal membrane-associated syntaxin 17 (Stx17) associates with synaptosome-associated protein 29 (SNAP29) and late endosome/lysosome-bound vesicle-associated membrane protein 7 (VAMP7) or VAMP8 to enable fusion between autophagosomes and lysosomes (129–132). However, in secretory autophagy, the autophagosomes do not fuse with lysosomes and instead fuse with the plasma membrane (either directly or indirectly after fusing with MVBs) (37, 119). In cells infected with poliovirus, coxsackievirus B3, or EV-D68, syntaxin 17 remains localized at the ER, excluded from the virus-shuttling autophagosome (37), and viral 3C proteases cleave SNAP29, thus preventing formation of the autophagosome–lysosome tethering complex (117, 133, 134). Secretory autophagy is critical for extruding mitochondria in red blood cells (135) and secreting lysozymes into the gut, among other cargo (136), but it is still unclear how the lysosomal and plasma membrane fusion pathways are differentiated in the cell.

The *Flaviviridae* DENV, Zika, and HCV may rely on secretory autophagy for some nonlytic egress, separate from the biosynthetic pathway (137–139). DENV proteins and full-length genomic RNA, Zika proteins and full-length genomic RNA, and clusters of DENV and Zika nucleocapsids have all been reported in secretory autophagosomes (or a chimeric secretory autophagosome–MVB fusion organelle) as well as the extracellular vesicles derived from them (106, 138, 140). Moreover, their rate of egress through secretory autophagy appears to be significantly faster, at least in the case of Zika, than their rate of egress via the biosynthetic pathway and is dependent on the Lyn and Ulk1 kinases, Rab GTPases, and SNARE complexes (138). HCV has been shown to activate autophagy and prevent autophagosome–lysosome fusion by downregulating autophagosome–lysosome fusion protein Stx17, suggesting a prominent role for secretory autophagy in HCV release (139, 141).

The *Arteriviridae* porcine reproductive and respiratory syndrome (PRRS) virus also appears to use both the biosynthetic pathway and a hybrid MVB–secretory autophagosome fusion organelle for egress, with the latter trafficking a cargo of full-length naked viral genomes mixed with viral proteins to the exterior. Notably, these extracellular vesicles are infectious to both naive, susceptible as well as nonsusceptible cells lacking CD163, the cognate PRRS receptor, suggesting direct fusion of the vesicles and delivery of the genomes (72).

In addition, two DNA-virus families have been shown to exploit secretory autophagy or a variation of it for nonlytic egress. Flomm et al. (142) used 3D correlative light electron microscopy to demonstrate that after nuclear egress, the *Herpesviridae* family member human cytomegalovirus nucleocapsids are captured within MVBs and/or secretory autophagosomes to generate multiviral bodies that are released into the extracellular environment inside vesicles. Members of the *Marseilleviridae* family of giant DNA viruses egress nonlytically from amoeba in extracellular vesicles of up to 5 μm in size and containing 30–50 virus particles. These vesicles originate from ER-derived double-membraned vesicles, likely secretory autophagosomes, which then fuse with the plasma membrane (143).

### 3.4. Plasma Membrane Budding

The plasma membrane is a well-known site of budding and egress for many viruses. The PS and phosphoinositide lipids that usually reside in the membrane inner leaflet play critical roles for the budding of many *Filoviridae*, *Paramyxoviridae*, and *Retroviridae* family members (91, 144–153). The Ebola virus and HIV matrix proteins VP40 and Gag bind PS and PI(4,5)P_2_, respectively, to dock, oligomerize, and induce membrane curvature before the final scission steps, which are coordinated by interaction of these matrix proteins with the ESCRT machinery (83, 153–156).

Likewise, rotaviruses and reoviruses also egress by budding from the plasma membrane (39, 41). In the case of rotaviruses, the viral nucleocapsids have been observed by correlative fluorescence scanning microscopy to egress en bloc by clustering in groups of 20–50 beneath the plasma membrane and budding in large vesicles 200–600 nm in diameter. Such vesicles have been observed to be shed in the feces of both humans and mice and can even comprise up to 50% of the total fecal viral pool (39). Similarly, parvoviruses, which egress nonlytically (157), are also found shed in stool in large vesicles 200–400 nm in diameter, suggesting these viruses bud from the plasma membrane, with each vesicle trafficking tens of virus particles (158). Clusters of BTV have been observed budding as extracellular vesicles from the plasma membrane in vitro and in vivo (159, 160). The BTV NS3 protein appears to be critical for regulating the budding in both mammalian and insect cells (40), and the budding involves the ESCRT machinery. NS3 contains an amphipathic helix that binds to the host exocytosis mediator annexin II protein complex (161) as well as two L-domain motifs responsible for binding the ESCRT machinery proteins TSG101 and NEDD4 at the plasma membrane (159).

### 3.5. Lysosomal Exocytosis

Lysosome fusion with the plasma membrane is termed lysosomal exocytosis. It is a calcium-regulated process that takes place in all eukaryotic cells and is important for plasma membrane repair, secretion of melanin pigments by melanocytes, bone resorption in osteoclasts, ATP release in the central nervous system, and antigen presentation and release of cell-killing enzymes in immune cells (162–165). Coronaviruses, including SARS-CoV-2, murine hepatitis virus (MHV), and porcine hemagglutinating encephalomyelitis virus, have all been reported to exploit lysosomal exocytosis to egress (166–171). Trafficking of these virus-loaded lysosomes to the plasma membrane is dependent on the small GTPases Arl8b and Rab7 (167, 168), and the fusion is dependent on synaptotagmin 7 (167). After replicating in ER-derived replication organelles, the nucleocapsids bud into the biosynthetic secretory pathway, becoming enveloped particles in the ER lumen. Subsequently, they traffic to lysosomes, either directly from the ER (172) or indirectly by transiting through the Golgi and TGN (163). Lysosomes are typically highly acidic (pH < 5), but coronaviruses disrupt acidification and avoid degradation by deacidifying the lysosomes through lysosomal targeting of pore-forming viral E and ORF3a proteins (167–169, 173–181). Lysosomal or lysosome–MVB fusion organelle–based nonlytic release pathways have also been proposed for some polyoma- and reoviruses (38, 182, 183).

In summary, the historical classification of viruses based on the presence or absence of a membrane around their nucleocapsids warrants reconsideration, as many previously characterized nonenveloped viruses egress by exploiting the same host membrane-trafficking egress pathways as enveloped viruses while minimizing cell lysis and cloaked in membranes.

## 4. HOW DO VIRUSES IN EXTRACELLULAR VESICLES INFECT?

The precise mechanisms by which exosomes and other such extracellular vesicles can carry out delivery of viral particles and/or naked nucleic acids are not fully understood. The PS lipids on the vesicle membrane outer leaflets facilitate tethering and subsequent endocytosis by binding to PS receptors like TIM-1 on cells (37, 39, 184, 185). Inside the endosome/lysosome, the vesicle membrane becomes degraded by enzymes including the lysosomal acid lipases and Niemann–Pick C1 cholesterol transporter, leading to the liberation of the viral nucleocapsids (185). These nucleocapsids then bind cognate receptors that are also present in the endosome. For polioviruses, these are the CD155 receptors and the nucleocapsids binding to them result in conformational changes in the receptors that lead to pore formation in the endosomal membrane and transfer of the poliovirus genomes en bloc across the endosomal membrane into the host cytosol (37). The transfer of the poliovirus genome also requires phospholipase A2 activity, which either initiates pore formation or stabilizes another pore (186). Lemon and colleagues (185) recently revealed using CRISPR screens that endosomal gangliosides, specifically the disialogangliosides GD3 and GD1, are essential coreceptors for HAV, and their binding leads to conformational changes in the nucleocapsid that possibly transfer the entire nucleocapsid(s) across the membrane prior to uncoating and release of the genomes into the cytosol. Notably, both the naked poliovirus and HAV, as well as their respective exosome/extracellular vesicle shuttled forms, use the same receptors for the final stages of genome or nucleocapsid transfer into the cytosol. For exosomes shuttling naked genomes, such as HCV, the exosomal membrane is thought to fuse directly with a target cell via the fusogenic activities of syncytin proteins embedded within the exosomal membrane. But the specific mechanism by which cells are targeted remains to be investigated.

## 5. VIRUSES CAN SPREAD CELL TO CELL WITHOUT EVER LEAVING THE CYTOPLASM

**Figure 3:**
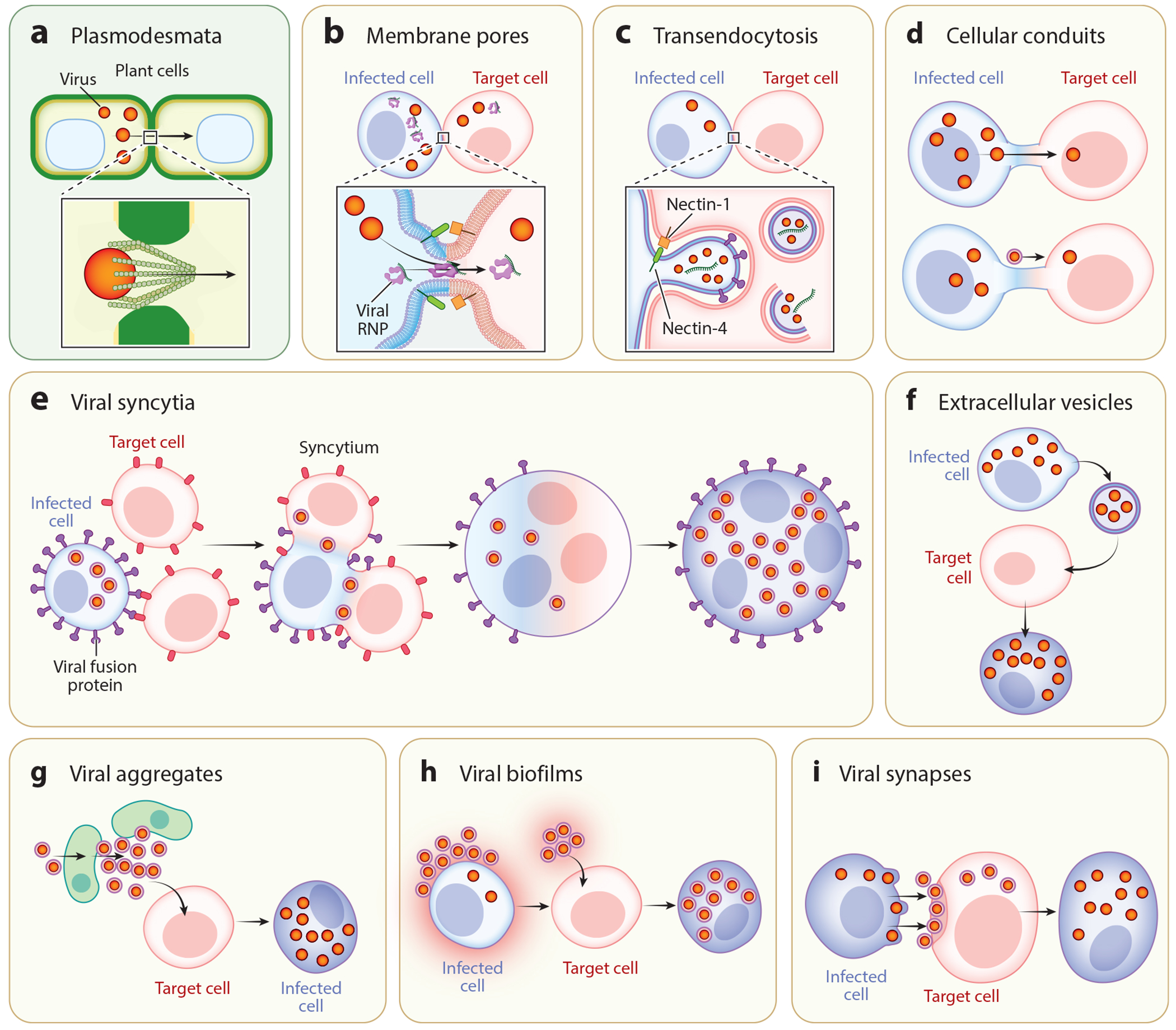
Nonlytic cell-to-cell egress and en bloc transmission. (*a*) Intercellular virus transmission is facilitated by plasmodesmata in plant cells. In animal cells, cell-to-cell viral transmission can occur through (*b*) membrane pores, (*c*) transendocytosis, (*d*) cellular conduits, (*e*) viral syncytia, (*f*) extracellular vesicles, (*g*) viral aggregates, (*h*) viral biofilms, and (*i*) virological synapses. Infected cells are shown in purple and target/uninfected cells are shown in pink. Abbreviation: RNP, ribonucleoprotein.

Membrane-cloaked egress is notable, as it enables viruses to leave the host cytoplasm and enter the extracellular milieu. This allows for long distance dissemination of viruses, including between tissues within the organism as well as among organisms, the latter through secretions (e.g., feces, aerosol, blood, saliva, skin, and breast milk) as well as vectors (mosquitos, ticks, etc.). Nonlytic transmission can also occur entirely through the intercellular movement of viruses without leaving the cytoplasm (Figure 3a–e), as described later in this section. These relatively short-distance viral transmission mechanisms are surprisingly widespread in the animal and plant virus worlds.

### 5.1. Egress Through Plasmodesmata and Membrane Pores

The presence of a cell wall forces many plant viruses to transmit through specialized intercellular channels called plasmodesmata (Figure 3a). This grants viruses the ability to transmit without ever strictly leaving the cytoplasmic compartment of the cells (51, 187). The infectious units that pass through plasmodesmata may consist of fully assembled nucleocapsids, naked viral genomes, or even viral replication complexes. The pores of the plasmodesmata are large enough (~50–60 nm in diameter) to accommodate plant viruses, which are typically ~30 nm in particle size, as well as supramolecular replication complexes, but they are gated by viral movement proteins, which interact with viral genomes and replicases to facilitate their spread (188–193).

Animal viruses also use membrane pores for nonlytic intercellular transmission. One notable example of this occurs with the *Paramyxoviridae* family member measles virus (MeV). In addition to budding into the extracellular space for transmission among primary human airway epithelia and between primary human myeloid cells and epithelial cells, MeV can spread through intercellular membrane pores (Figure 3b) (194, 195). Like plant viruses, MeV replication complexes, so-called infectious centers, traffic between neighboring cells through pores of ~250 nm. This involves the reorganization of actin filaments through the interaction of the actin-binding protein afadin and the MeV receptor Nectin4 to facilitate viral transfer through the membrane (194). Furthermore, using recombinant green fluorescent protein–tagged MeV and live-cell imaging techniques, the ribonucleoprotein (RNP) complex of MeV has been shown to spread to adjacent cells by moving along the circumapical F-actin rings, which are required to facilitate the movement of RNP through the lateral membrane pores (196).

### 5.2. Egress Through Transendocytic Uptake and Tunneling Nanotubes

Another mode of egress reported for MeV involves the cytoplasmic contents of infected cells, including infectious centers and encapsidated MeV genomes, being endocytosed by neighboring cells through Nectin-1/Nectin-4–adherens complexes (197). This process has been termed transendocytosis or Nectin-elicited ectoplasmic transfer (NECT) (Figure 3c). Notably, NECT appears to be a novel transmission mechanism for crossing the tropism barrier, as it enables MeV to spread from epithelial cells, which express Nectin-4, to primary neurons, which express Nectin-1.

A variation of the above mechanism involves viruses spreading among cells via nanotubes (Figure 3d), tunneling nanotubes (TNTs), or bridging conduits (198–201). The cytoplasmic compartments of cells can be connected to one another via TNTs, which have typical inner diameters of 20–700 nm and lengths up to 100 μm. The Rho GTPase cdc42 and the atypical motor protein Myosin 10 are both required for TNT biogenesis, and cargo is trafficked through them via actin-based motility. HIV was one of the first viruses reported to utilize TNTs for transmitting genome-loaded capsids, viral RNA, and the proteins Gag and Nef between macrophages and T cells, as well as between macrophages and B cells (202–204). Retroviruses are not unique in using TNTs: Many other virus families have also been reported to use them for nonlytic intercellular spread, including *Alpha*- and *Gammaherpesvirinae* (205–207), *Pneumoviridae* (208), *Poxviridae* (209), *Orthomyxo*- and *Paramyxoviridae* (210, 211), *Arteriviridae* (212), *Flaviviridae* (213), *Sedoreoviridae* (214), *Arenaviridae* (215), *Picornaviridae* (216), and *Coronaviridae* (217). Viruses such as HIV and SARS-CoV-2 can also surf along the extracellular surface of the TNTs via direct contact with surface molecules such as heparan sulfate proteoglycans (217–219). The extracellular surface of TNTs can provide multiple docking sites for viruses and thus enable the long-distance transfer of multiple viral particles to distant cellular targets.

### 5.3. Egress Through Syncytia

Viral fusion proteins appearing on the cell surface can induce cell-to-cell fusion, generating multinucleate cells termed syncytia (Figure 3e). This enables newly replicated virions, naked viral genomes, and the replication machinery to spread intercellularly without lysing cells. Syncytia formation is a multistep sequential process that involves binding of the viral fusion protein expressed on the plasma membrane of the infected cell with the receptor on the adjacent cell; activation of fusion protein, leading to its conformational change and insertion into the target cell membrane; and finally, merging of both plasma membranes (220, 221). Syncytia formation has been observed in cells infected with *Coronaviridae* (SARS-CoV-2, MERS, MHV) (222–228), *Pneumoviridae* (229–231), *Paramyxoviridae* (232), *Herpesviridae* (233, 234), and *Retroviridae* family members (235, 236). SARS-CoV-2 extensively induces syncytia formation, and multinucleated pneumocytes are frequently observed in lung samples of infected patients (237, 238). SARS-CoV-2-infected cells express viral spike proteins on their surface, which enables fusion with neighboring cells that express the ACE-2 receptor on their surface. Moreover, TMPRSS2, a serine protease that is known to enhance infection, increases syncytia formation by processing ACE-2 and spike protein, leading to fusion (223, 225). Multiple different reovirus and rotavirus strains have also been reported to express fusion-associated small transmembrane proteins that trigger cell-to-cell fusion and form syncytia (239–241).

Collectively, the above examples highlight the variety of mechanisms employed by viruses to transmit themselves, not only without lysing the host cells but even without traversing to the extracellular milieu, to avoid likely encounters with host antibodies.

## 6. NONLYTIC EGRESS MECHANISMS OF ARCHAEAL AND BACTERIAL VIRUSES

Nonlytic egress is not unique to the animal and plant virus world. Many archaeal viruses employ nonlytic egress mechanisms to preserve host archaeal cell integrity and allow for continuous viral progeny production (Figure 1) (55, 57). One such mechanism employed by *Fuselloviridae* is budding, which utilizes machinery like the ESCRT machinery of eukaryotic cells (56). Another used by *Sulfolobaceae* relies on virus-associated pyramids (VAPs) to egress (54). VAPs are seven-faceted pyramidal outward protrusions of proteins that integrate into and span both the plasma membrane and the surrounding paracrystalline S-protein layer of archaeal cells. Once the VAPs reach a size of ~150 nm, the tip of the VAP opens, along with the seams of the pyramidal facets, allowing the newly formed archaeal virions to pass from the cytosol, across the membrane and S layer, and into the extracellular space before the VAP is closed up (55, 57).

While the majority of bacteriophages egress lytically, there are notable exceptions (52, 242) of functional significance for our review of egress for animal and plant viruses. One of the unique features of the *Inoviridae* family, which infect Gram-negative bacteria, is the establishment of chronic infections during which phages are continuously and nonlytically released from the bacterial cell. To do so, *Inoviridae* take advantage of the bacterial secretion machinery to exit the outer membrane (243, 244). Additionally, a few phage-encoded proteins facilitate egress through the bacterial inner membrane by forming pores through which the viral DNA passes. Since the viral coat proteins are assembled on the inner bacterial membrane, viral assembly occurs while the viral genome is passing through the pores (52, 242). Further, the viral proteins, which are homologous to bacterial proteins of the type II secretion system and type IV pili, facilitate viral exit by forming a barrel-like structure on the outer membrane (Figure 1) (53). The generation of *Inoviridae* progeny from the infected bacterium is a continuous process and does not lead to cell death.

## 7. ADVANTAGES OF NONLYTIC EGRESS: QUALITY CONTROL

In contrast to the indiscriminate release of viruses from lysed cells, viruses that are packaged in extracellular vesicles for nonlytic egress pathways (Figure 3f) enable the selective transmission of only infectious particles to the extracellular milieu. For example, genome-devoid particles, which are made in large numbers during infection, are excluded from egress, and only genome-containing poliovirus nucleocapsids are captured in secretory autophagosomes and released from cells in extracellular vesicles (37, 120). There also appears to be less variation in the genetic makeup of the viruses captured by autophagosomes (245). This may be a consequence of poliovirus replication, assembly, and capture into autophagosomes occurring in spatial proximity to one another on the surface of ER-derived replication organelles (37, 120, 246). Poliovirus nucleocapsids have been reported to contain sequences that are recognized by the autophagy cargo receptors p62/SQSTM1 (247), which could facilitate the replication-competent genomes in being assembled and packaged for egress.

Another example of this kind of quality control can be seen in studies of rotavirus transmission. The rotavirus VP4 nucleocapsid protein must be proteolytically cleaved into VP5 and VP8 for rotaviruses to be activated and infectious (248). Prior to the discovery of extracellular vesicles trafficking rotavirus out of cells nonlytically (39), rotaviruses were thought to egress in the gastrointestinal tract by lysis, and stool proteases were assumed to catalyze VP4 cleavage and activation. However, analysis of the vesicle-contained rotaviruses shed in stool revealed that they were already in the infectious activated (VP5/VP8) state (39). This suggested that VP4 proteolytic cleavage was likely taking place either during packaging into or postbudding of the extracellular vesicle. Notably, the free rotavirus nucleocapsids present in stool were much lower in infectivity than equivalent nucleocapsid loads inside vesicles. Furthermore, as would be expected from exposure to stool proteases, the free pool of nucleocapsids exhibited significant proteolytic degradation (39). The vesicle-contained pools, in contrast, remained intact and protected. Overall, through nonlytic egress, viruses set up a process of transmission with better quality control (i.e., with the assembly and packaging of fully formed infectious viruses) compared to the messier process of lytic egress.

## 8. ADVANTAGES OF NONLYTIC EGRESS: EN BLOC TRANSMISSION

**Figure 4:**
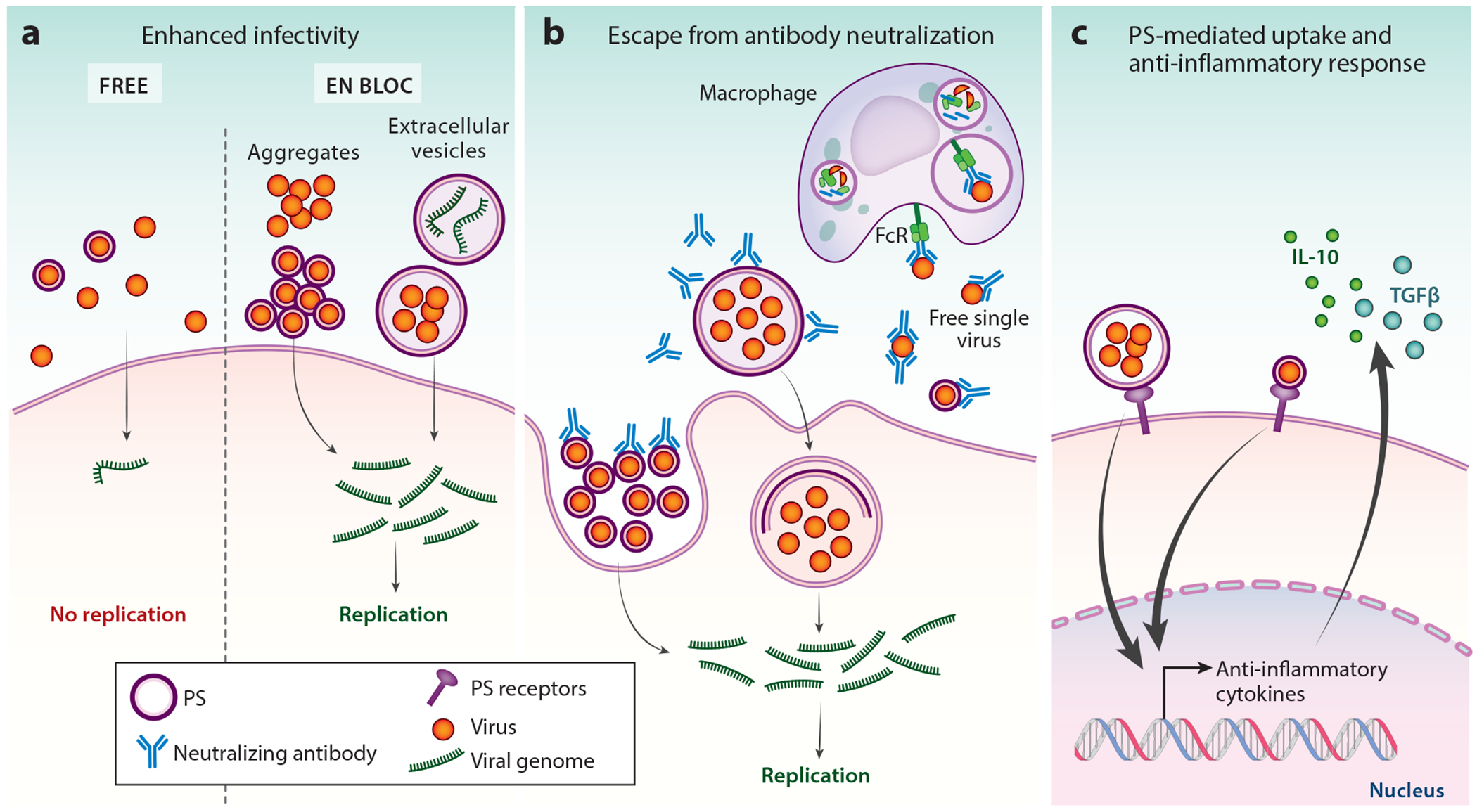
Advantages of en bloc viral transmission. (*a*) Viruses traveling en bloc inside vesicles or in aggregates enter cells with high multiplicity of infection, allowing them to overcome replication barriers and establish infection; free viral particles do not transfer enough genomes into cells to overcome replication barriers. (*b*) En bloc transmission of viruses protects viruses from antibody neutralization. (*c*) Virus uptake and suppression of innate immune responses are increased by viruses traveling en bloc in PS lipid vesicles or by enveloped viruses containing PS lipids in their membrane. Abbreviations: FcR, antibody Fc receptor; PS, phosphatidylserine.

A significant consequence of nonlytic egress is that it provides a path for multiple infectious units, whether they may be nucleocapsids, naked genomes, or a combination of both, to transmit collectively, through en bloc transmission (37, 73, 249). As described later in this section, viruses carry out en bloc transmission in a variety of ways, including by being transported as populations inside extracellular vesicles (Figure 3f), by piggy-backing in multiples on bacteria (Figure 3g), by aggregating on biofilms (Figure 3h), and by being transmitted at high multiplicities across intercellular channels, virological synapses, TNTs, and syncytia (Figure 3a–e, i). En bloc transmission enhances infectivity by increasing the multiplicity of infection, which can lead to more rapid production of viral proteins and genomes and the ability to overcome host innate defenses, as well as providing opportunities for cooperation among genetic variants (Figure 4a).

### 8.1. En Bloc Egress and Transmission Through Extracellular Vesicles

As described in Section 3, many viruses exploit membrane-trafficking pathways to egress cloaked in membrane and en bloc to the extracellular environment (37, 39, 245, 250). Poliovirus (37), coxsackievirus (37, 116), rhinovirus (37), rotavirus (39), norovirus (39, 250), reovirus (41), parvoviruses (157), BTV (160), polyomaviruses (98, 100), cytomegalovirus (142), marseillevirus (143), HCV (103, 109), Langat virus (105), DENV (107), and many others all nonlytically egress as viral particle populations and/or naked genomes inside extracellular vesicles (Figure 3f) (73, 107, 251, 252). This form of en bloc transmission, which has been observed to take place in cell culture, in infected animal models, and in clinical samples (saliva, stool, etc.), enhances infectivity and virulence (37, 39, 41, 98, 102, 108, 143, 160, 183). As detected by in situ RNA hybridization methodologies, inoculated extracellular vesicles containing viral populations deliver their viral genome cargo en bloc into new host cells (37). When cells or animals are inoculated with vesicles containing multiple viral particles, the virus infectivity is significantly higher compared with the equivalent quantity of free viral particles, suggesting that viral input thresholds exist for successful replication and that viruses need to enter cells in sufficient numbers to overcome host innate defenses and replicate. Moreover, en bloc delivery may promote cooperative interactions among viral quasispecies (73, 201, 253). Surprisingly, vesicles containing multiple rotavirus, norovirus, and parvovirus particles had been observed in stool specimens as early as the 1980s, but their significance in viral transmission was overlooked, as free viral pools in the stool were thought to be the main agents of infectivity (158).

### 8.2. En Bloc Transmission Through Viral Aggregates, Bacterial Scaffolds, and Biofilms

Independently of extracellular vesicles, viral particles can also enhance their multiplicity of infection by aggregation. Conventional enveloped viruses like coronavirus, HIV, and vesicular stomatitis virus (VSV), as well as poliovirus, rotavirus, norovirus, and adenovirus, form nucleocapsid aggregates in stool, the latter group in nonenveloped extracellular states due to untimely release from lytic cells (Figure 3g) (254–262). Different biotic and abiotic factors can influence the formation of viral aggregates. For example, physicochemical parameters such as pH, ionic strength, composition, and temperature can affect viral aggregation by altering the physicochemical surface properties of the virus (262). Protein–lipid interactions and protein-driven interactions have been seen to positively influence VSV aggregate formation in purified stock and in saliva, respectively. A saliva protein, fibrinogen gamma protein, has also been identified as promoting VSV aggregation in saliva (263).

A similar strategy is used for en bloc transmission in viral biofilms, where clusters of newly egressed virus particles are embedded into a glycosylated protein matrix on the surface of cells (Figure 3h) (200). For instance, human T-cell leukemia virus (HTLV) particles are stored in clusters on the surface of infected CD4^+^ T lymphocytes (264). These clusters, rich in carbohydrates, are held together by virally induced extracellular matrix components like collagen and agrin, a heparan sulphate proteoglycan, along with cellular linker proteins such as tetherin and galectin-3 (264, 265). These extracellular viral assemblies facilitate en bloc transmission of viruses to other cells by promoting rapid cell adherence. Viral biofilms are thought to be the major contributor to HTLV-1 infection, as their removal reduces infectivity by approximately 80% (264, 265). Extracellular HTLV-1 biofilms share striking similarities with bacterial biofilms and potentially offer advantages in terms of both enhanced spread and evasion of the immune response.

Bacteria in the gastrointestinal tract have also been observed to act as scaffolds and bind multiple viruses (Figure 3g). These bacterial scaffolds, like the extracellular vesicles, aggregates, and biofilms, increase the multiplicity of infection and the genetic diversity of viruses entering cells and enhance the overall fitness of the viral population (253, 266).

#### 8.2.1. En bloc transmission through virological synapses.

Yet another strategy for en bloc transmission involves virological synapses (Figure 3i), which enable high concentrations of viral particles to be transmitted between closely juxtaposed cells, in a process similar to that which occurs at neural and immunological synapses (264, 267–269). The biogenesis of these synapses and their role in egress has been best studied for retroviruses, specifically HTLV and HIV; with these viruses, reorientation of the microtubule organizing center forces viral components including Gag and RNA to cluster at cell junctions and polarizes viral budding toward the synapse between the infected and uninfected cell (270). This form of viral spread is pivotal for retroviral dissemination in vivo (271, 272) and is less sensitive to neutralizing antibodies compared to the transmission of free viral particles (198, 273–275).

#### 8.2.2. En bloc transmission through syncytia.

Viruses can induce syncytia (i.e., multinucleated cells resulting from cell fusion) to transfer viral particles, genomes, or a combination of both en bloc (Figure 3e). Subacute sclerosing panencephalitis is a serious and potentially fatal brain inflammation that can occur after a MeV infection. Notably, MeV neuropathogenesis and brain persistence appears to be highly dependent on the en bloc transmission of viral variants (276, 277). The virus’s fusogenic F protein is known to undergo hypermutation, leading to cell-to-cell membrane fusion and syncytia formation. However, the mutant F proteins can induce membrane fusion only when coexpressed with the wild-type fusion proteins, with neither having the ability to induce cell fusion independently. Thus, MeV can persist and spread in the nervous system only via the transmission en bloc of multiple viral particles (wild type and mutated) (276).

## 9. ADVANTAGES OF NONLYTIC EGRESS: IMMUNE SYSTEM EVASION

Nonlytic egress has significant advantages for evading the immune system. First, the membrane cloaks of extracellular vesicles largely protect viral cargo from antibodies (278, 279) (Figure 4b). Note that vesicle membranes may themselves contain embedded viral nonstructural and structural proteins that can potentially be targeted by some antibodies, although myeloid cells generally need high densities of targeted antibodies for opsonization. Apart from extracellular vesicles, viral aggregates and biofilms may also achieve the same protection against neutralizing antibodies by physically restricting their access to many of the virions inside the aggregates (265, 280, 281).

Secondly, PS lipids found exposed on the outer membrane leaflets of enveloped single viral particles or extracellular vesicles transporting viral clusters or naked viral genomes (37, 282) bind PS receptors in the TAM (Tyro3, Axl, and Mer), TIM (T-cell immunoglobulin and mucin domain–containing proteins), and CD300 families, and trigger release of anti-inflammatory cytokines and chemokines including IL-10 and TGFβ (283). In addition, PS binding to PS receptors facilitates viral entry into cells, a strategy utilized by vaccinia, cytomegalovirus, HIV, DENV, Ebola, coronaviruses, HAV, poliovirus, norovirus, and rotavirus (73, 283, 284). Retroviruses, coronaviruses, and HAV all utilize the TIM family (TIM1, 3, and 4) whereas the calicivirus MNV-1 uses the CD300lf family of PS receptors for infection (39, 283, 285). Masking viral PS lipids with the PS-binding protein annexin V or blocking the PS receptors with antibodies significantly impedes the infectivity of HIV, poliovirus, norovirus, and many others (Figure 4c) (37, 39, 282, 286, 287).

## 10. VIRUSES EXPLOIT MULTIPLE DIFFERENT NONLYTIC EGRESS PATHWAYS

As has likely become obvious from the above discussions, viruses take advantage of multiple membrane-trafficking pathways to egress without lysis. For example, SARS-CoV-2 can egress and transmit to the extracellular environment (and thereby other hosts) by lysosomal exocytosis (167) as well as spread between neighboring cells by forming syncytia (225). It has even been reported that extracellular vesicles may be shed and carry naked full-length SARS-CoV-2 genomes to other cells at distant sites (251). HCV and the flaviviruses Zika, DENV, West Nile, and Langat all egress not only by being secreted from the biosynthetic pathway as individual particles but also by being shed en bloc as full-length naked genomes in extracellular vesicles via the endo/lyso/autophagosomal exocytic pathways. Similarly, retroviruses rely on both direct plasma membrane budding and the endo/lyso/autophagosomal exocytic pathway for egress, the latter mostly in phagocytic cells (89). Perhaps the most versatile virus is MeV, which nonlytically egresses through plasma membrane budding, intercellular membrane pores, transendocytosis, and syncytia formation. Note that transendocytosis and syncytia formation can lead to more rapid transmission than spread by cell-free virus particles. Moreover, having multiple different infectious forms that egress by different routes may be an evolutionary strategy to maximize propagation, with some infectious forms enhancing the multiplicity of infection, others better evading the immune system, and yet other forms enhancing genetic diversity.

## 11. CONCLUSIONS

An effective virus is one that does not typically kill its host but instead makes use of the living cell with its organelles, energy metabolism, and protein and lipid building blocks to sustain and propagate itself. During the process of egress from one host to spread to another, many eukaryotic viruses leave behind intact cells. Intact cells may not only sustain additional virus production but also delay activation of the immune response. As described in this article, viruses employ multiple different nonlytic strategies of egress and transmission including inducing syncytia, using intercellular pores, and most notably, using host membrane-trafficking pathways.

Many outstanding questions remain. The extent of nonlytic egress in the world of viruses remains to be fully explored. In the Supplemental Material, we lay out experimental steps to first determine whether a virus egresses nonlytically and second to identify the egress pathway it utilizes (Supplemental Figure 1). How viruses choose a nonlytic egress pathway and whether and how this depends on cell type are not known. The stage of the viral life cycle may also affect selection of the egress pathway due to the dynamic nature of viral infections, with time-dependent increases in viral genomes and proteins gradually reorganizing and even eliminating host organelles and trafficking pathways (246). Different egress pathways may also influence which tissues are next infected by the viruses. Naked viral genomes packaged in extracellular vesicles, in contrast to individual free viral particles, may enable the viruses to bypass the restrictions of receptor presence and infect a broader swath of tissues. Indeed, the mechanisms by which extracellular vesicles target and deliver their viral cargoes to various tissues are still very poorly understood and remain to be investigated. En bloc egress through extracellular vesicles or aggregates also increases the likelihood of a successful next infection compared to free virus particles, and transmission by syncytia or intercellular channels enhances the odds of viral spread by hiding from the immune system. More work needs to be done in vivo to investigate tissue tropism and the role of the immune cells in cell killing and clearing of lysed cells,.

In conclusion, the egress pathways taken are critical elements that determine the success or failure of viral transmission. We hope the discussion here, illustrating the convergence of so many viral families, previously classified as vastly different from one another, on the same nonlytic egress and transmission strategies, may prompt investigation into alternative views of viral relationships and evolution.

## Supplementary Material

Supplementary Material
