# Supplementary material for: Nonlytic Egress and Transmission in the Virus World
Source: Annu Rev Biochem. Author manuscript; Available in PMC 2026 Jun 22. (PMC13285319; doi:10.1146/annurev-biochem-052521-120140)
Supplement: Supplementary Material [file NIHMS2184423-supplement-Supplementary_Material.pdf]

Downloaded from www.annualreviews.org. NIH: National Institutes of Health Library (ar-154384) IP: 156.40.252.14 On: Fri, 05 Jun 2026 16:17:38

SUPPLEMENTAL MATERIAL: METHODOLOGY FOR DETERMINING NON-LYTIC EGRESS

i. Quantifying kinetics of cell lysis versus viral egress

A small molecule exclusion assay, with molecules such as trypan blue that are unable to permeate across the plasma membrane, is the first step in determining if a virus of interest egresses nonlytically (**Supplemental Figure 1*i***). In this simple assay:

1. Cells are inoculated with the virus of interest at a high multiplicity of infection for enough time to allow entry of the virus into the cell but not start replication.
2. Cells are then washed free of the virus and the media replaced with fresh cell culture medium.
3. At time intervals thereafter, the supernatant is collected along with the cells.
4. Viral content of the supernatant is quantified by PCR.
5. Harvested cells are incubated with a cell impermeable small molecule such as Trypan Blue according to manufacturer's instructions and the percentage of trypan blue negative/positive cells are counted with a cell counter.
6. The quantity of virus in the supernatant is plotted against the quantity of trypan blue negative cells to determine if viral egress took place prior to cell lysis.

ii. Isolating membrane-cloaked viruses

If the small molecule exclusion assays above suggest nonlytic viral egress, then next step will be to isolate the membrane-cloaked viruses from cell culture medium using one of the methods below (**Supplemental Figure 1*ii***). Note that these methods can also be utilized to isolate membrane cloaked viruses from bodily fluids and secretions including feces, blood, saliva, milk and cerebrospinal fluid.

By pull down of PS-lipids: Most if not all enveloped viruses and extracellular vesicles transporting viruses have phosphatidylserine lipids in their outer membrane leaflets. PS-binding proteins such as Annexin V (Miltenyi Biotec) or TIM4 coupled to magnetic beads (MagCapture™ Exosome isolation kit, FujiFilm) can be used to selectively capture and isolate membrane-cloaked viruses which can then be eluted from these beads and characterized. The characterization can include measuring the viral content using PCR or Western Blotting;

morphology via negative stain electron microscopy; morphology via light microscopy using lipophilic membrane intercalating fluorescent dyes such as PKH (Millipore Sigma) and ExoBrite™(Biotium); and testing the infectivity of the isolated vesicles by inoculating them into cell culture or animals (39). The advantage of the PS-based isolation method is that it can be used to isolate vesicles from very small volumes (<200 microliters) and the purity of the isolated material. The drawbacks of this method are: not high yield (compared to centrifugal isolation) and won't separate virus filled vesicles from nonvirus containing vesicles.

By pull down of exosomal membrane markers. Using antibodies targeted to extracellular vesicle membrane proteins is another strategy for isolation. There are a number of commercial sources for exosome purification via antibody based pull down (ThermoFisher, Luminous Biosciences, Promega). Extracellular vesicles derived from MVBs typically are enriched in tetraspanins including CD9, CD63 and CD81; plasma membrane derived vesicles often are enriched in CD98; and secretory autophagosome derived vesicles may contain all these proteins as autophagosomal membranes mostly originate from the ER. Compared to the PS-based pull-down method, this has similar drawbacks with an additional major drawback that it relies on the antibody targeted membrane protein being enriched in the membrane cloaked viruses.

By ultracentrifugation: This method relies on centrifugation to isolate virus filled vesicles based on their size, shape and density. Infected samples are initially centrifuged at a lower speed to remove cells and debris and then ultracentrifuged at gradually higher speeds to pellet down vesicles of different sizes. Generally, vesicles larger than 200 nm in diameter will come down at 10,000–20,000g whereas vesicles less than 200 nm in diameter will come down at 100,000g. While yield with this method is high, the drawbacks of this method are: precipitate may bringdown aggregates of naked viruses, cellular debris etc. along with vesicles; centrifugation at high speeds may break or damage the vesicles; and large quantities of starting material are needed for sequential centrifugation A variation of this method is to centrifuge through a Nycodenz or glycerol gradient which may help separate the virus containing vesicles from other nonvirus containing vesicles and aggregates (69).

iii. Visualizing extracellular vesicles containing viruses (**Supplemental Figure 1*iii***)

Microscopy is an indispensable tool in virology, providing detailed visual insights into the complex processes involved in viral egress. The ability to visualize the exit of viruses from host cells has significantly advanced our understanding of viral life cycles, host-pathogen

Downloaded from www.annualreviews.org. NIH: National Institutes of Health Library (ar-154384) IP: 156.40.252.14 On: Fri, 05 Jun 2026 16:17:38

interactions, and the development of antiviral strategies.

Fluorescence Microscopy: Different fluorescent microscopes such as wide-field fluorescence microscope, confocal microscope, or super-resolution microscope are widely used in studying different aspects of viral life cycle. Using fluorescent lipophilic dyes (see above) or by fluorescent protein tagged viral markers, in conjunction with high-resolution light-based imaging techniques, such as structured illumination microscopy (SIM), photoactivated localization microscopy (PALM) and stochastic optical reconstruction microscopy (STORM), one can visualize the membrane-cloaked viruses that are released. In addition, these, as well as standard confocal imaging, can identify the cellular pathways exploited by the viruses for nonlytic release. Tagging viral and/or host cell components with fluorescent proteins or staining them with fluorescent antibodies/dyes can allow detailed visualization and analysis of the viral life cycle. In addition, identifying antibodies that can distinguish the fully assembled viral particles from viral particle components (individual capsid subunits or envelope proteins) can be very useful to immunostain and show the relative location of newly assembled viral particles in the cell. One starting point to find such antibodies is to screen known neutralizing antibodies as often these antibodies recognize complex epitopes formed on assembled viral particles (Chen et al. 2015; Ghosh et al. 2020). By acquiring images at different time points and taking time-lapse live images of these fluorescently tagged/stained viruses and cellular organelle markers, one can begin to shed light into the cellular pathways exploited by the virus in question. With the more commonplace availability of super-resolution light microscopes, we are in an era where we have or are near the resolution advantage of electron microscopy with minimal processing of the sample that allows preservation of membranes.

Electron Microscopy (EM): Transmission electron microscopy (TEM) and Scanning electron microscopy (SEM) are powerful high resolution imaging tools that can provide insight into the virus-host interface. But as sample preparation typically requires harsh treatment conditions that can strip away membranes, these methods should not be solely relied upon to assess the membrane-cloaking state of the virus in question. Another method that can be used on cell culture medium isolates, that is relatively less harsh in sample preparation and disruption of membranes yet still enhances the contrast of viral particles to provide high resolution information, is negative staining. Here the isolates from the cell culture medium are dropped on carbon coated copper grids, fixed and stained with aqueous 0.5% w/v uranyl acetate solution or

Downloaded from www.annualreviews.org. NIH: National Institutes of Health Library (ar-154384) IP: 156.40.252.14 On: Fri, 05 Jun 2026 16:17:38

NanoVan before imaging under the electron microscope.

Correlative Light and Electron Microscopy (CLEM): Correlative Light and Electron Microscopy (CLEM) is a technique that combines light and electron microscopy to identify and localize viral and host proteins at high resolution. First, live or fixed virus-infected cells are plated on gridded coverglass and imaged using fluorescently tagged live-cell reporters or antibodies, respectively, against viral and host proteins. The positions of the fluorescent structures are noted due to the grid. The samples are then processed for electron microscopy and aligned and overlaid with the light microscopy data. CLEM has been successfully used to study the egress pathways of poliovirus, rotavirus, herpes simplex and Influenza A viruses. While this is a powerful method, the same concerns hold regarding sample preparation for the electron microscopy.

Downloaded from www.annualreviews.org. NIH: National Institutes of Health Library (ar-154384) IP: 156.40.252.14 On: Fri, 05 Jun 2026 16:17:38

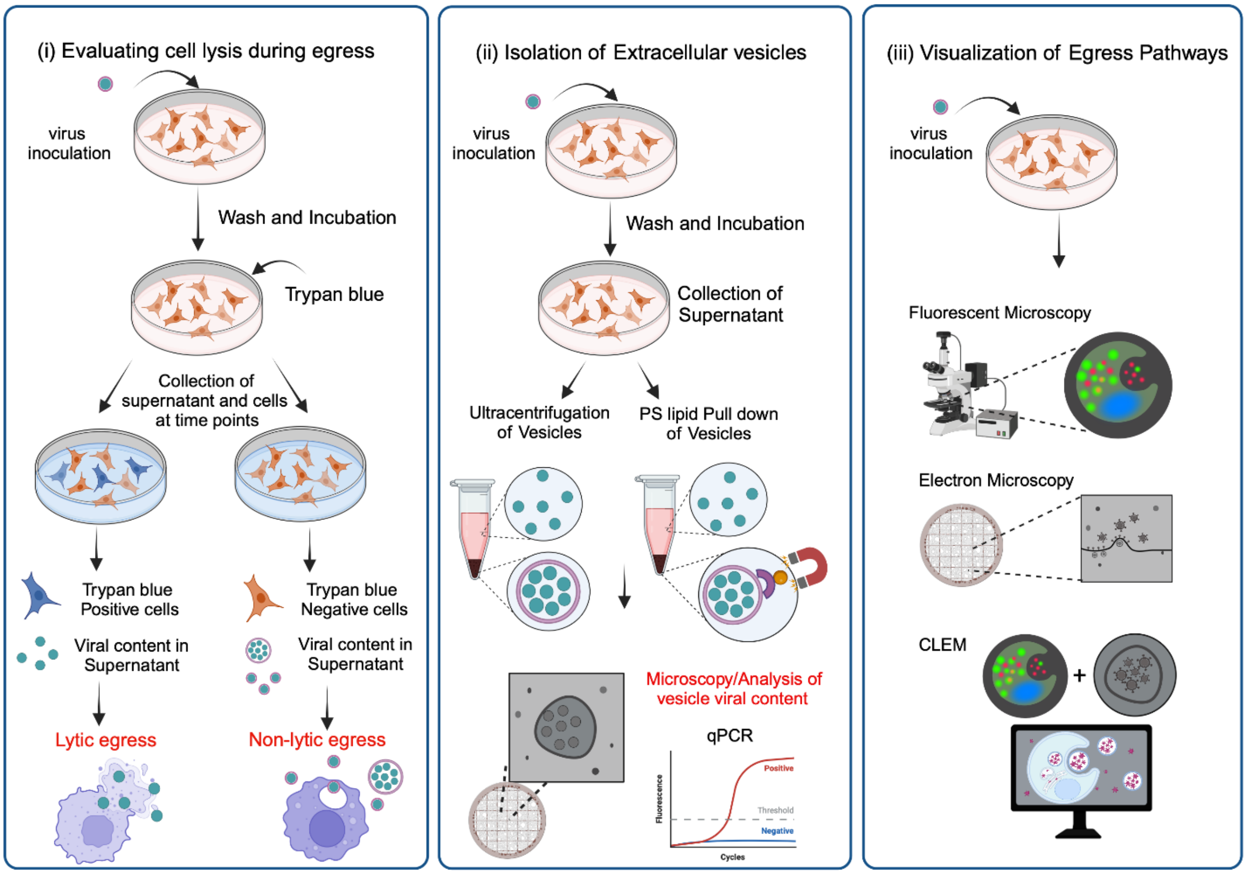

Supplemental Figure 1: Schematic representation of methodologies for evaluating viral egress: (i) Evaluation of cell lysis during egress, (ii) Isolation of virus containing extracellular vesicles (EV), and (iii) visualization of virus containing EVs and viral egress pathways. Created in BioRender. Panigrahi, M. (2025) https://BioRender.com/r63x406

Downloaded from www.annualreviews.org. NIH: National Institutes of Health Library (ar-154384) IP: 156.40.252.14 On: Fri, 05 Jun 2026 16:17:38
